# Negative Impact of Fear of COVID-19 on Health-Related Quality of Life Was Modified by Health Literacy, eHealth Literacy, and Digital Healthy Diet Literacy: A Multi-Hospital Survey

**DOI:** 10.3390/ijerph18094929

**Published:** 2021-05-06

**Authors:** Minh H. Nguyen, Thu T. M. Pham, Kien T. Nguyen, Yen H. Nguyen, Tien V. Tran, Binh N. Do, Hung K. Dao, Huu C. Nguyen, Ngoc T. Do, Tung H. Ha, Dung T. Phan, Khue M. Pham, Linh V. Pham, Phuoc B. Nguyen, Hoai T. T. Nguyen, Thinh V. Do, Dung T. Ha, Hung Q. Nguyen, Huong T. M. Ngo, Manh V. Trinh, Thuy T. T. Mai, Nhan P. T. Nguyen, Anh L. Tra, Thao T. P. Nguyen, Kien T. Nguyen, Chyi-Huey Bai, Tuyen Van Duong

**Affiliations:** 1 International Ph.D. Program in Medicine, College of Medicine, Taipei Medical University, Taipei 110-31, Taiwan; d142108015@tmu.edu.tw; 2 Faculty of Public Health, Hai Phong University of Medicine and Pharmacy, Hai Phong 042-12, Vietnam; phamminhthu.ytcc@gmail.com (T.T.M.P.); pmkhue@hpmu.edu.vn (K.M.P.); 3 School of Public Health, College of Public Health, Taipei Medical University, Taipei 110-31, Taiwan; 4 President Office, Can Tho University of Medicine and Pharmacy, Can Tho 941-17, Vietnam; ntkien@ctump.edu.vn; 5 Department of Physiology, Faculty of Medicine, Can Tho University of Medicine and Pharmacy, Can Tho 941-17, Vietnam; 6 Department of Pharmacology and Clinical Pharmacy, Can Tho University of Medicine and Pharmacy, Can Tho 941-17, Vietnam; nhyen@ctump.edu.vn; 7 Department of Pharmacy, Can Tho University of Medicine and Pharmacy Hospital, Can Tho 941-17, Vietnam; 8 Ph.D. Program in School of Pharmacy, College of Pharmacy, Taipei Medical University, Taipei 110-31, Taiwan; 9 Department of Infectious Diseases, Vietnam Military Medical University, Hanoi 121-08, Vietnam; tientv@vmmu.edu.vn (T.V.T.); nhubinh.do@vmmu.edu.vn (B.N.D.); 10 Director Office, Military Hospital 103, Hanoi 121-08, Vietnam; 11 Division of Military Science, Military Hospital 103, Hanoi 121-08, Vietnam; 12 Director Office, Bac Ninh Obstetrics and Pediatrics Hospital, Bac Ninh 161-23, Vietnam; daokhachung2000@yahoo.com; 13 Director Office, E Hospital, Hanoi 113-08, Vietnam; bacsyhuu@gmail.com; 14 Department of Thoracic and Cardiovascular Surgery, E Hospital, Hanoi 113-08, Vietnam; 15 Nursing Office, E Hospital, Hanoi 113-08, Vietnam; dothingocbve@gmail.com; 16 Director Office, General Hospital of Agricultural, Hanoi 125-16, Vietnam; hahuutung.200564@gmail.com; 17 Faculty of Nursing, Hanoi University of Business and Technology, Hanoi 116-22, Vietnam; phanthidzungvd@gmail.com; 18 Nursing Office, Thien An Obstetrics and Gynecology Hospital, Hanoi 112-06, Vietnam; 19 President Office, Hai Phong University of Medicine and Pharmacy, Hai Phong 042-12, Vietnam; 20 Department of Pulmonary & Cardiovascular Diseases, Hai Phong University of Medicine and Pharmacy Hospital, Hai Phong 042-12, Vietnam; pvlinh@hpmu.edu.vn; 21 Director Office, Hai Phong University of Medicine and Pharmacy Hospital, Hai Phong 042-12, Vietnam; 22 Director Office, Kien An Hospital, Hai Phong 046-09, Vietnam; nguyenbatuankiet@gmail.com; 23 Training and Direction of Healthcare Activity Center, Kien An Hospital, Hai Phong 046-09, Vietnam; hoaibvka@gmail.com; 24 Director Office, Bai Chay Hospital, Quang Ninh 011-21, Vietnam; dovanthinhhscc@gmail.com; 25 Nursing Office, Bai Chay Hospital, Quang Ninh 011-21, Vietnam; hadungbvbc@gmail.com; 26 Director Office, Quang Ninh Obstetrics and Pediatrics Hospital, Quang Ninh 011-24, Vietnam; bshungbvqn@gmail.com; 27 Nursing Office, Quang Ninh Obstetric and Pediatric Hospital, Quang Ninh 011-24, Vietnam; ngomaihuong.hl@gmail.com; 28 Director Office, Quang Ninh General Hospital, Quang Ninh 011-08, Vietnam; trinhmanhqnsyt@gmail.com; 29 Nursing Office, Quang Ninh General Hospital, Quang Ninh 011-08, Vietnam; maithanhthuy68@gmail.com; 30 General Planning Department, Da Nang Oncology Hospital, Da Nang 506-06, Vietnam; bsnhanbvub@gmail.com; 31 Department of Physiotherapy and Rehabilitation, Da Nang University of Medical Technology and Pharmacy, Da Nang 502-06, Vietnam; hanitra2630@gmail.com; 32 Health Management Training Institute, University of Medicine and Pharmacy, Hue University, Thua Thien Hue 491-20, Vietnam; nguyenthiphuongthao@hueuni.edu.vn; 33 Department of Health Economics, Corvinus University of Budapest, 1093 Budapest, Hungary; 34 Department of Health Education, Faculty of Social Sciences, Behavior and Health Education, Hanoi University of Public Health, Hanoi 119-10, Vietnam; ntk1@huph.edu.vn; 35 Department of Public Health, College of Medicine, Taipei Medical University, Taipei 110-31, Taiwan; 36 School of Nutrition and Health Sciences, Taipei Medical University, Taipei 110-31, Taiwan

**Keywords:** fear of COVID-19, health-related quality of life, health literacy, eHealth literacy, digital healthy diet literacy

## Abstract

Background: The COVID-19 pandemic has been disseminating fear in the community, which has affected people’s quality of life, especially those with health problems. Health literacy (HL), eHealth literacy (eHEAL), and digital healthy diet literacy (DDL) may have potential impacts on containing the pandemic and its consequences. This study aimed to examine the association between the fear of COVID-19 scale (FCoV-19S) and the health-related quality of life (HRQoL), and to examine the effect modification by HL, eHEAL, and DDL on this association. Methods: A cross-sectional study was conducted in 11 hospitals across Vietnam from 7 April to 31 May 2020. Data were collected on 4348 outpatients, including demographic characteristics, HL, eHEAL, DDL, FCoV-19S, and HRQoL. Multiple linear regression and interaction models were used to explore associations. Results: Patients with higher FCoV-19S scores had lower HRQoL scores (unstandardized coefficient, B = −0.78, *p* < 0.001). HL (B = 0.20, *p* < 0.001), eHEAL (B = 0.24, *p* < 0.001), and DDL (B = 0.20, *p* < 0.001) were positively associated with higher HRQoL scores. The negative impact of FCoV-19S on HRQoL was significantly attenuated by higher eHEAL score groups (from one standard deviation (SD) below the mean, B = −0.93, *p* < 0.001; to the mean, B = −0.85, *p* < 0.001; and one SD above the mean, B = −0.77, *p* < 0.001); and by higher DDL score groups (from one SD below the mean, B = −0.92, *p* < 0.001; to the mean, B = −0.82, *p* < 0.001; and one SD above the mean, B = −0.72, *p* < 0.001). Conclusions: eHealth literacy and digital healthy diet literacy could help to protect patients’ health-related quality of life from the negative impact of the fear of COVID-19 during the pandemic.

## 1. Introduction

The COVID-19 pandemic has been placing unprecedented challenges and burdens on the economic, health, and political systems of the affected countries [1,2,3]. The numbers of new infections and deaths are increasing, and there is no sign of control [4]. Therefore, the COVID-19 pandemic has been significantly affecting people’s health and well-being around the world.

Preventive measures have been implemented worldwide to control COVID-19 transmissions such as lockdowns, social distancing, mask-wearing, and handwashing [5]. However, these measures have also caused a wide range of negative consequences such as a lack of social connection, mental health problems, and lifestyle changes [6,7]. Thus, people’s psychological and physical health have seriously been affected, resulting in a deterioration in the health-related quality of life (HRQoL). Therefore, it is crucial to determine the risk and protective factors affecting the health-related quality of life during a pandemic to develop early and effective interventions to improve the HRQoL.

The COVID-19 pandemic is still rapidly spreading with record numbers of new infections and deaths, leading to an increased fear of virus transmission in the community. Fear may cause adverse effects such as lifestyle changes, delays in healthcare access, and mental health problems such as depression or even suicide [8,9]. Thus, it can affect people’s quality of life, especially high-risk groups such as the elderly and people with health problems [10,11].

During the time of social distancing and limited connection with others, people spent more time interacting on social media. Social media has become a popular means for communication, entertainment, and accessing health information [12,13]. However, social media has also been rapidly spreading false and inaccurate information about COVID-19, which may raise concerns and fears in the public [14]. eHealth literacy (eHEAL) is the capacity to seek, find, understand, and appraise basic health information from Web-based sources and to apply the knowledge acquired to solve health problems [15]. Therefore, eHealth literacy and health literacy (HL) have potential roles in dealing with misinformation and containing the spread of COVID-19 in the community [16,17]. Besides, improving HL and eHEAL could help people engage in active behaviors such as healthy diets, physical activities, and adherence to preventive measures during the pandemic [18], which could further improve the health status and well-being [19].

Healthy diets have shown beneficial effects on the enhancement of immune systems and health outcomes during the COVID-19 pandemic [20,21,22]. Moreover, a well-balanced and adequate diet can improve the immune response to viral infections (e.g., COVID-19) [23], and reduces the risk of chronic diseases [24,25]. In a recent study, healthy dietary intake was a protective factor that could reduce the risk of depression during the COVID-19 lockdown period [26]. Thus, eating a healthy diet is crucial to help people stay fit, which improves their quality of life during the pandemic. However, unbalanced eating habits are rising worldwide [27,28], including in Vietnam [29]. During the pandemic, governments have implemented preventive measures (e.g., lockdowns and social distancing), which has negatively impacted people’s mental health and living habits, including diet and eating behaviors. Therefore, it is essential to improve the knowledge about healthy and proper diets during the COVID-19 crisis. Digital healthy diet literacy (DDL) is the ability to seek, understand, appraise, and apply healthy diet information from electronic sources to improve healthy eating habits [30]. A previous study conducted in Vietnam indicated that DDL was positively associated with healthier eating habits [30]. Hence, by improving healthy diets, DDL may have a potential effect in enhancing the quality of life during a pandemic. However, to date, no studies have evaluated that relationship. Therefore, assessing the relationship between DDL and HRQoL is critical to providing timely suggestions for public health interventions and future studies.

This study was conducted to explore the association of the fear of COVID-19 (FCoV-19S), HL, eHEAL, and DDL with the health-related quality of life (HRQoL), and to examine the effect modification by HL, eHEAL, and DDL on the association between FCoV-19S and HRQoL among outpatients in 11 hospitals across Vietnam.

## 2. Materials and Methods

### 2.1. Study Design and Settings

We conducted a cross-sectional study between 7 April and 31 May 2020 at 11 hospitals in Vietnam, including nine hospitals in the Northern area, one hospital in the Central area, and one hospital in the Southern area.

The study was reviewed and approved by the Institutional Ethical Review Committee of Hanoi University of Public Health, Vietnam (IRB No. 133/2020/YTCC-HD3).

### 2.2. Study Sample and Data Collection

During a pandemic, all resources and budgets in hospitals are re-arranged for epidemic prevention. In addition, it has been urgent to provide timely evidence for interventions to improve the quality of life for the outpatient population during a pandemic, so we used the convenience sampling method, which is a rapid method and minimizes financial and human burdens. Patients who visited the outpatient departments (OPD) at the selected hospitals were recruited into the study. Eligible participants were those aged between 18 and 85 years, who were able to read and write Vietnamese, and who agreed to join the survey. Patients with any emergency conditions (e.g., stroke, respiratory failure, and serious injuries), dementia, psychotic disorder, or blindness were excluded. Finally, the overall sample of 4348 respondents was collected and analyzed in our study. The distribution of recruited participants from each hospital is presented in Table 1.

At each selected hospital, research assistants (medical staff and medical students) were trained in 2 sessions: (1) data collection; (2) infection control (e.g., mask-wearing, washing hands, and physical distancing) using guidelines of the World Health Organization [31].

The research assistants contacted and consecutively invited outpatients who presented at the OPDs to join the survey. Patients voluntarily participated and signed the informed consent form before conducting the investigation. We used a self-administered questionnaire to collect the data. People who visited hospitals could either pick a printed self-administered questionnaire or scan the QR code using their smartphone to link to the online questionnaire. In most of the hospitals, the participants used printed self-administered questionnaires to complete the survey. During the survey period, research assistants constantly supervised and assisted the participants in completing the survey. It took 15–20 min to complete each survey. Finally, the data were cleaned and coded for analysis.

### 2.3. Instruments and Measurements

#### 2.3.1. Health-Related Quality of Life

The health-related quality of life (HRQoL) was assessed with the short form (SF-36) [32], which is widely used in Vietnam studies [33,34]. The self-administered SF-36 consisted of 36 questions evaluating the quality of life with 8 health aspects (e.g., vitality, physical function, bodily pain, general health, physical role function, emotional role function, and mental health). The scoring method was provided in the user guidelines [35]. The overall score varied between 0 and 100, with a higher score indicating a better HRQoL.

#### 2.3.2. Fear of COVID-19

The fear of COVID-19 was evaluated using a 7-item fear of COVID-19 scale (FCoV-19S), which has been developed and validated in Iran [36] and in Vietnam [37], with satisfactory reliability and validity. The Cronbach’s alpha of FCoV-19S in this study was 0.92. Respondents rated their perception about the susceptibility to COVID-19 infectability on a 5-point Likert scale from 1 (strongly agree) to 5 (strongly disagree). The responses were summed up, and the total score was between 7 and 35, with a higher score representing a higher level of fear of COVID-19.

#### 2.3.3. Health Literacy and Digital Healthy Diet Literacy

Health literacy (HL) was measured with a short-form health literacy questionnaire (HLS-SF12), which was widely used for studies in Asia [38] and in Vietnam [34,39,40,41]. The Cronbach’s alpha of HLS-SF12 in the current study was 0.95.

Digital healthy diet literacy (DDL) was measured using a DDL-4 questionnaire. This questionnaire has been developed and validated in Vietnam, and the reliability and validity were satisfactory [30]. The Cronbach’s alpha of DDL-4 was 0.96 in this study.

The HLS-SF12 and DDL-4 questionnaires consisted of 12 and 4 items, respectively. Patients responded to the questionnaires on the 4-point Likert scale regarding the difficulty in conducting each item ranging from 1 (very difficult) to 4 (very easy). The HL and DDL scores were transformed into a unified index between 0 and 50, with a higher score indicating a better HL or DDL. The formula has been described in a previous study [30].

#### 2.3.4. eHealth Literacy

The participants’ eHealth literacies were measured with an 8-item eHealth literacy scale (eHEALS), which has been validated and used in Vietnam, and the reliability and validity were satisfactory [18]. In our study, the Cronbach’s alpha of eHEALS was 0.96. Respondents rated their degree of agreement with 8 items about their experiences in health-related information processing on the internet on a 5-point Likert scale ranging from 1 (strongly disagree) to 5 (strongly agree). The total score was between 8 and 40, with a higher eHEALS score indicating a better eHealth literacy.

#### 2.3.5. Participants’ Characteristics

The patients’ demographic information was collected, including age (years), sex (men vs. women), marital status (never married vs. ever married), education degree (secondary school or below, high school, or college/university or higher), occupational status (unemployed/dependents vs. employed), ability to pay for medication (very difficult to very easy), and social status (patients self-assessed their position in the society in terms of education, occupation, and income at 3 degrees of low, middle, and high). Participants reported their body weight (kg) and height (cm). Body mass index (BMI, kg/m^2^) was calculated and classified into 2 groups (normal weight (BMI < 25.0) vs. overweight/obese (BMI ≥ 25.0)).

Respondents were screened for the suspected COVID-19 symptoms (S-COVID-19-S) at the time of the survey, including common symptoms (fever, cough, and dyspnea) and less common symptoms (myalgia, fatigue, sputum production, confusion, headache, sore throat, rhinorrhea, chest pain, hemoptysis, diarrhea, and nausea/vomiting) [42]. Participants were classified as having suspected COVID-19 symptoms if they had any of these symptoms. Respondents self-reported their comorbidities using the Charlson Comorbidity Index items [43]. We classified the participants into 2 groups (with vs. without comorbidity).

#### 2.3.6. Lifestyle Changes

Patients provided their information about current lifestyle behaviors in comparison to those before the epidemic. Smoking, drinking, and physical activity were assessed on a five-response scale: never, stopped, less, unchanged, and more. Eating behavior was reported on a three-response scale: healthy, unchanged, healthier. According to WHO recommendations, to stay fit during the pandemic, people should maintain or improve their living habits, such as staying physically active, eating a healthy diet, quitting tobacco, and avoiding alcohol [44]. The “unchanged or healthier” diet and “unchanged or more” physical activity were considered as positive behaviors, and “unchanged or more” smoking or drinking were considered as negative behaviors. Therefore, patients’ responses were categorized into two groups: “never/stopped/less” vs. “unchanged or more” for smoking, drinking, and physical activity, and “less healthy” vs. “unchanged or healthier” for healthy eating.

### 2.4. Statistical Analysis

First, the distribution of studied variables was presented with a number (n), percentage (%), mean, and standard deviation (SD) appropriately. Next, the t-test or one-way ANOVA test was used to explore group differences in HRQoL scores. Thirdly, the associations of FCoV-19S, HL, eHEALS, and DDL with HRQoL were analyzed in different individual models using simple and adjusted linear regression analysis. In addition to age and gender, other factors correlated with HRQoL in the bivariate analysis (*p* < 0.20) were adjusted in multivariate analysis. To eliminate the multicollinearity, we analyzed correlations between adjusted factors using Spearman’s correlation. If two factors showed a moderate correlation (*rho* ≥ 0.30), one representative factor was put into the adjusted models. These results are given in Tables S1–S3 in Supplementary Materials. Finally, we examined the modification effects of HL, eHEALS, and DDL on the association between FCoV-19S and HRQoL using interaction models. If the interaction term was statistically significant, we conducted the simple slope analysis to visualize the interaction effect. To perform simple slope analysis, three variables, FCoV-19S, eHEALS, and DDL, were standardized into Z-scores with a mean of zero and a standard deviation of one. The plots were drawn by evaluating the values of HRQoL for three values of both the continuous independent variable FCoV-19S and continuous moderator variables (eHEALS or DDL), including Z = 1 (one standard deviation above the mean), Z = 0 (the mean), and Z = −1 (one standard deviation below the mean), and creating three lines to indicate the effect of FCoV-19S on the HRQoL at the three values of eHEALS or DDL. The *p*-value < 0.05 was defined as significant. We used the IBM SPSS Version 20.0 for data analysis (IBM Corp, Armonk, NY, USA).

## 3. Results

### 3.1. Demographic Characteristics

The average age of the sample was 42.8 years. The mean scores of HL, eHEALS, DDL, and FCoV-19S were 26.5, 27.9, 25.9, and 20.6, respectively. Of 4348 respondents, 62% were women, 82.2% ever married, 49.3% had college/university or higher degrees, 89.1% were employed, 37.5% felt it was easy to pay for treatments, 12.6% were overweight or obese, and 40.3% had suspected COVID-19 symptoms. In terms of the changes in health-related behaviors as compared to those before the pandemic, the proportion of participants with “unchanged or healthier” eating behaviors was 92.5%, while the figures for “unchanged or more” smoking, “unchanged or more” drinking alcohol, and “unchanged or more” physical activity were 8.1%, 7.4%, 42.2%, respectively (Table 2).

### 3.2. Associations of HL, eHEALS, DDL, and FCoV-19S with HRQoL

In the bivariate models, age, marital status, education, occupation, ability to pay for medications, social status, BMI, comorbidity, S-COVID-19-S, physical activity, and healthy eating were associated with HRQoL at *p* < 0.20 (Table S1 in Supplementary Materials). To avoid multicollinearity, the correlations among the confounders were examined. Moderate correlations were found between age and education (*rho* = −0.34); between the ability to pay for medications and social status (*rho* = 0.30); between comorbidity with S-COVID-19-S (*rho* = 0.50), HL (*rho* = −0.38), eHEALS (*rho* = −0.38), and DDL (*rho* = −0.37); between S-COVID-19-S and HL (*rho* = −0.34), eHEALS (*rho* = −0.35), and DDL (*rho* = −0.31) (Table S2 in Supplementary Materials). Therefore, age, gender, marital status, occupation, social status, BMI, physical activity, and healthy eating were put into the adjusted models. After adjusting for confounders, multiple linear regression models showed that patients with a high level of FCoV-19S had a lower HRQoL score (unstandardized regression coefficient, B = −0.78, 95% CI −0.87, −0.70, *p* < 0.001). eHEALS (B = 0.24, 95% CI 0.17, 0.32, *p* < 0.001) and HL (B = 0.20, 95% CI 0.15, 0.25, *p* < 0.001) were positively associated with HRQoL. A higher DDL score was found to be associated with a higher HRQoL (B = 0.18, 95% CI 0.14, 0.22, *p* < 0.001; Table 3).

### 3.3. Effect Modification by eHEALS and DDL on the Association between FCoV-19S and HRQoL

**Figure 1:**
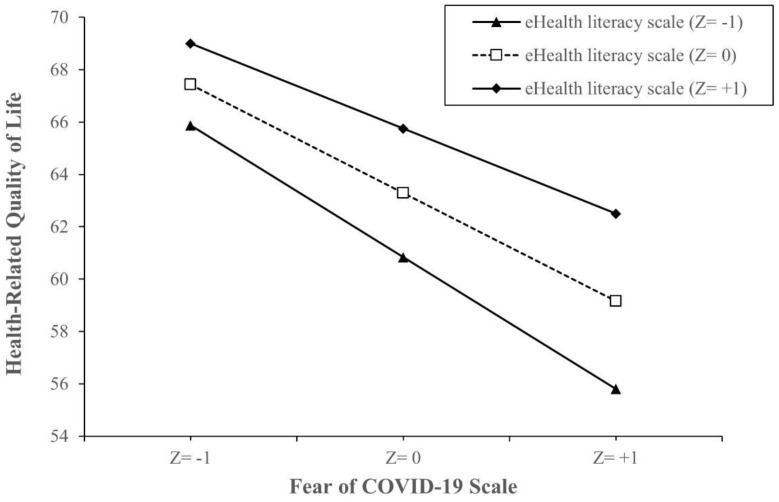
Simple slope plot of interaction between eHealth literacy and fear of COVID-19 on health-related quality of life (N = 4348). Note: Z = −1, one SD below the mean; Z = 0, the mean; Z = +1, 1 SD above the mean. Z-score is the number of SD above or below the mean. Abbreviations: SD, standard deviation.

Table 4 shows the interaction model between eHealth literacy and the fear of COVID-19. Compared to patients with the lowest FCoV-19S score and eHEALS score, those with the lowest eHEALS score and 1 FCoV-19S-score increment had lower HRQoL scores (B = −1.18, 95% CI −1.51, −0.85, *p* < 0.001), while those with 1 eHEALS-score increment and 1 FCoV-19S-score increment had higher HRQoL scores (B = 0.01, 95%CI 0.01, 0.02, *p* = 0.034) (Table 4). Simple slope analysis showed that when eHEALS is higher, the impact of FCoV-19S on HRQoL becomes weaker. This impact was attenuated by higher eHEALS groups from the value of 1 SD below the mean (B = −0.93, 95% CI −1.06, −0.81, *p* < 0.001) to the mean (B = −0.85, 95% CI −0.94, −0.76, *p* < 0.001), and the value of 1 SD above the mean (B = −0.77, 95% CI −0.88, −0.66, *p* < 0.001). The model is visualized in Figure 1.

**Figure 2:**
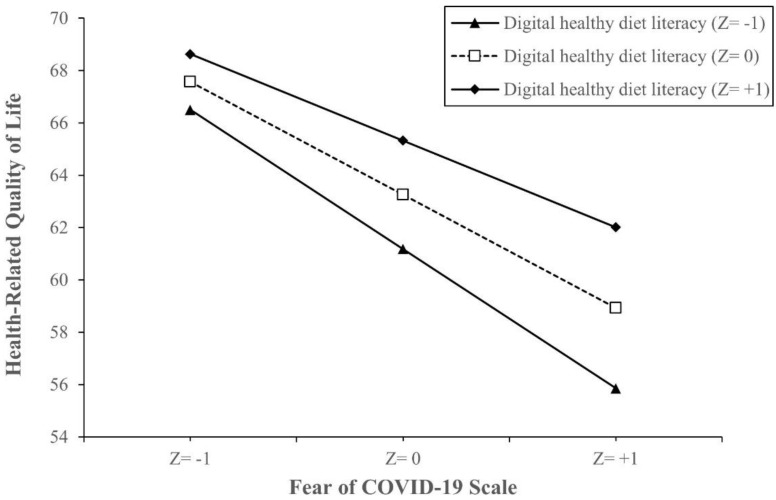
Simple slope plot of interaction between digital healthy diet literacy and fear of COVID-19 on health-related quality of life (N = 4348). Note: Z = −1, one SD below the mean; Z = 0, the mean; Z = +1, 1 SD above the mean. Z-score is the number of SD above or below the mean. Abbreviations: SD, standard deviation.

In the interaction model between digital healthy diet literacy and fear of COVID-19, as compared to patients with the lowest FCoV-19S score and DDL score, those with the lowest DDL score and 1 FCoV-19S-score increment had lower HRQoL scores (B = −1.02, 95% CI −1.22, −0.82, *p* < 0.001), while those with 1 DDL-score increment and 1 FCoV-19S-score increment had higher HRQoL scores (B = 0.01, 95% CI 0.00, 0.02, *p* = 0.016) (Table 4). Simple slope analysis showed that when DDL is higher, the association between FCoV-19S and HRQoL becomes weaker. The impacts of FCoV-19S on HRQoL were lowered by higher DDL score groups from the value of 1 SD below the mean (B = −0.92, 95% CI −1.04, −0.79, *p* < 0.001), to the mean (B = −0.82, 95% CI −0.91, −0.73, *p* < 0.001), and the value of 1 SD above the mean (B = −0.72, 95% CI −0.83, −0.63, *p* < 0.001). The model is visualized in Figure 2.

The result did not show the significant effect modification by HL on the association between FCoV-19S and HRQoL in the multivariate model (Table 4).

## 4. Discussion

In this study, people with higher FCoV-19S scores had lower HRQoL scores. This finding was consistent with a previous study among the general population in China [45]. Several reasons could also explain this association. First, fears of COVID-19 transmission can cause mental health problems such as anxiety, depression, or even suicide [46,47], which may negatively affect the HRQoL [48]. Secondly, patients may be delayed access to healthcare systems due to fears of disease infection, especially in patients with chronic diseases [9]. Besides, during the COVID-19 crisis, the fear of COVID-19 may cause sedentary behaviors and negative lifestyle changes such as unhealthy diets, inactive physical activities, or substance abuse [37,49]. All of these factors may worsen physical health and well-being [50]. Therefore, it is essential to develop timely public health strategies to mitigate the concerns and fears related to COVID-19, which may improve the quality of life among patients.

In the current study, higher scores of health literacy and eHealth literacy were associated with higher HRQoL scores. These findings were similar to results from previous studies [19,51]. Besides, higher health literacy and eHealth literacy were also positively associated with a healthy diet and physical exercise [18,52], which may further improve well-being.

Our study showed that eHealth literacy could alleviate the adverse effect of FCoV-19S on health-related quality of life, whereas health literacy was not effective in the model of interaction analyses. These results might be explained by the fact that during the COVID-19 pandemic, people spent more time on the Internet and social media for communicating and finding information [49]. They also faced a wide range of disinformation and misinformation about COVID-19, causing confusion and fears [53,54]. Therefore, people with a high degree of eHealth literacy may better find reliable sources and appraise online health information, thereby reducing the FCoV-19S and improving the HRQoL [17].

In addition, as social media and news websites have rapidly become essential tools for communicating and disseminating information for the community [12,13], the critical role of eHealth literacy has been highlighted during the pandemic. That is why health literacy could enhance the quality of life in our study, but it could not mitigate the harmful effect of FCoV-19S on HRQoL. However, the role of health literacy has still been crucial both before and during the pandemic [55].

Therefore, timely and comprehensive interventions need to be developed to boost health literacy and eHealth literacy for communities, which would further improve the quality of life amid the pandemic, especially in patients with fears of COVID-19. A previous study showed that a higher fear of COVID-19 score was associated with a lower health literacy [37]. Thus, to improve health literacy and e-health literacy, governments and health organizations need to inform the community through mass media with timely, accurate, transparent, and plain information about the COVID-19 epidemic, such as COVID-19 symptoms, means of transmission, and effective prevention measures. Another critical approach is providing accessible, reliable, and trusted health information sources (e.g., credible websites or forums) that assist individuals in enhancing their beneficial health knowledge and in avoiding COVID-19-related misinformation during the pandemic. As smart devices are becoming more popular, technological solutions also need to be applied (e.g., patient portals, telemedicine, and health-related mobile applications) to help healthcare providers connect and consult more effectively with patients, which may make a significant contribution to enhancing patient’s health literacy [56].

We found that people with higher DDL scores had higher HRQoL scores. In the interaction model, a higher DDL score significantly weakened the negative impact of FCoV-19S on HRQoL. These findings could be explained by people with better DDL having a higher likelihood of adherence to healthier eating behaviors during the pandemic [30]. Another study conducted among the adult population in the Netherlands also indicated that higher food literacy was positively associated with a healthy diet [57]. Meanwhile, previous studies have suggested that the adherence to healthy dietary patterns could enhance the health-related quality of life [58,59,60,61]. Balanced and nutritious diets help to improve health status, reduce the risk of chronic diseases [62,63], and potentially reduce the risk of complications and severe conditions of COVID-19 [25]. Better diet quality was found to be associated with a lower risk of depression [26,64]. Thus, better DDL may help patients with COVID-19-related fears have a better dietary intake, thereby strengthening their physical and mental health, and their quality of life may gradually improve. This suggests that DDL is a critical issue that should be highlighted for intervention promotion to improve the HRQoL, especially in the digital age with too many inaccurate information sources on the internet.

Our study had some limitations. First, as the survey was conducted amid the pandemic, both research assistants and studied respondents were highly vulnerable to COVID-19 infection. Thus, all interviewers were requested to adhere to the national guidelines about infection prevention throughout the data collection procedure. Fortunately, no new confirmed cases were reported during the period of data collection [65]. Second, a causal relationship could not be drawn from the cross-sectional study. Thirdly, our discussion focused on explaining the relationship between DDL and HRQoL through improving healthy eating behaviors. However, the relationship between DDL and healthy eating behaviors could be modified by several factors (e.g., income, occupation, social status, and food insecurity). Therefore, future studies need to confirm our findings. Finally, the findings should be generalized to the outpatient population with caution due to the convenient sampling method. However, as our study was conducted with a relatively large sample size, the results may produce timely evidence for developing public health interventions to improve the health-related quality of life, especially in those with fears of COVID-19 during the pandemic.

## 5. Conclusions

In this study, the fear of COVID-19 was negatively associated with the health-related quality of life. In contrast, health literacy, eHealth literacy, and digital healthy diet literacy were positively associated with the health-related quality of life. eHealth literacy and digital healthy diet literacy can help to mitigate the negative effects of the fear of COVID-19 on the health-related quality of life. Strategical public health interventions should be promoted to improve eHealth literacy and digital healthy diet literacy, which would further enhance the health-related quality of life, especially among patients with fears of COVID-19.

## Figures and Tables

**Table 1:** Study sample at 11 hospitals across Vietnam.

Geographic Location	Hospitals	Studied Participants
**Northern area**		
Ha Noi city		
	1. Military Hospital 103	501
	2. E Hospital	183
	3. General Hospital of Agricultural	300
Hai Phong city		
	4. Hai Phong University of Medicine and Pharmacy Hospital	490
	5. Kien An Hospital	492
Quang Ninh province		
	6. Bai Chay Hospital	364
	7. Quang Ninh Obstetrics and Pediatrics Hospital	280
	8. Quang Ninh General Hospital	309
Bac Ninh city		
	9. Bac Ninh Obstetrics and Pediatrics Hospital	500
**Central area**		
Da Nang city		
	10. Da Nang Oncology Hospital	421
**Southern area**		
Can Tho city		
	11. Can Tho University of Medicine and Pharmacy Hospital	508
**Total**		4348

**Table 2:** Characteristics and health-related quality of life among outpatients (N = 4348).

Variables	Total (N = 4348)	HRQoL	
	n (%)	Mean (SD)	*p* *
Age (years), mean (SD)	42.8 (16.7)		
Age groups			<0.001
<60	3412(78.5)	65.8 (17.6)	
≥60	936 (21.5)	53.8 (15.6)	
Gender			0.454
Women	2694 (62.0)	63.0 (18.1)	
Men	1654 (38.0)	63.5 (17.5)	
Marital status			<0.001
Never married	772 (17.8)	69.7 (15.6)	
Ever married	3560 (82.2)	61.8 (18.1)	
Education attainment			<0.001
Secondary school or below	1007 (23.2)	58.2 (18.3)	
High school	1196 (27.5)	61.1 (18.2)	
College/university or higher	2139 (49.3)	66.8 (16.7)	
Occupation			<0.001
Unemployed	474 (10.9)	56.5 (19.5)	
Employed	3874 (89.1)	64.0 (17.5)	
Ability to pay for treatments			<0.001
Very or fairly difficult	2712 (62.5)	60.9 (17.7)	
Very or fairly easy	1626 (37.5)	67.1 (17.5)	
Social status			<0.001
Low	921 (21.2)	59.6 (20.6)	
Middle or high	3419 (78.8)	64.2 (16.9)	
BMI, kg/m^2^			0.156
Normal weight (BMI < 25.0)	3791 (87.4)	63.3 (18.0)	
Overweight/obese (BMI ≥ 25.0)	546 (12.6)	62.2 (17.2)	
Suspected COVID-19 symptoms **			<0.001
No	2595 (59.7)	65.5 (17.2)	
Yes	1753 (40.3)	59.7 (18.3)	
Comorbidity			<0.001
No	3094 (71.2)	64.6 (17.8)	
Yes	1254 (28.8)	59.6 (17.6)	
Smoking tobacco ***			0.329
Never, stopped, or smoke less	3994 (91.9)	63.1 (18.0)	
Unchanged or smoke more	354 (8.1)	64.1 (16.4)	
Drinking alcohol ***			0.918
Never, stopped, or drink less	4015 (92.6)	63.2 (18.1)	
Unchanged or drink more	321 (7.4)	63.3 (15.6)	
Physical activity ***			<0.001
Never, stopped, or exercise less	2515 (57.8)	58.5 (17.5)	
Unchanged or exercise more	1833 (42.2)	69.7 (16.3)	
Healthy eating ***			<0.001
Less healthy	325 (7.5)	55.6 (12.8)	
Unchanged or healthier	4002 (92.5)	63.8 (18.1)	
Health literacy, mean (SD)	26.5 (10.5)		
eHealth Literacy Scale, mean (SD)	27.9 (6.9)		
Digital healthy diet literacy, mean (SD)	25.9 (12.2)		
Fear of COVID-19 Scale, mean (SD)	20.6 (5.4)		

Abbreviation: HRQoL, health-related quality of life; SD, standard deviation; BMI, body mass index; COVID-19, coronavirus disease 2019. * Result of t-test or one-way analysis of variance (ANOVA) test. ** The suspected COVID-19 symptoms including common symptoms (fever, cough, and dyspnea) and less common symptoms (myalgia, fatigue, sputum production, confusion, headache, sore throat, rhinorrhea, chest pain, hemoptysis, diarrhea, and nausea/vomiting). *** Patients were asked about changes in their current health-related behaviors as compared to those before the COVID-19 pandemic.

**Table 3:** Associations of fear of COVID-19, health literacy, eHealth literacy, and digital healthy diet literacy with health-related quality of life using multiple linear regression models (N = 4348).

Variables	HRQoL			
	Model 1 **		Model 2 ***	
	B (95% CI)	*p*	B (95% CI)	*p*
Fear of COVID-19 Scale, 1-score increment *	−0.83 (−0.93, −0.74)	<0.001	−0.78 (−0.87, −0.70)	<0.001
Health literacy, 1-score increment *	0.41 (0.36, 0.46)	<0.001	0.20 (0.15, 0.25)	<0.001
eHealth Literacy Scale, 1-score increment *	0.50 (0.42, 0.57)	<0.001	0.24 (0.17, 0.32)	<0.001
Digital healthy diet literacy, 1-score increment *	0.38 (0.33, 0.42)	<0.001	0.18 (0.14, 0.22)	<0.001

Abbreviation: HRQoL, health-related quality of life; B, unstandardized regression coefficient; CI, confidence interval; COVID-19, coronavirus disease 2019. * The associations of the Fear of COVID-19 Scale, health literacy, eHealth literacy, and digital healthy diet literacy with health-related quality of life were analyzed in separate individual models. ** Model 1: Simple linear regression model. *** Model 2: Adjusted for age, gender, marital status, occupation, social status, BMI, physical activity, and healthy eating.

**Table 4:** Interactions of the fear of COVID-19 with health literacy, eHealth literacy, and digital healthy diet literacy on health-related quality of life (*n* = 4348).

Interactions	HRQoL			
	Model 1 *		Model 2 **	
	B (95% CI)	*p*	B (95% CI)	*p*
**Interaction between FCoV-19S and HL**				
FCoV-19S, 1-score increment	−1.16 (−1.39, −0.92)	<0.001	−1.00 (−1.21, −0.78)	<0.001
HL, 1-score increment	0.22 (0.05, 0.38)	0.008	0.11 (−0.05, 0.26)	0.177
FCoV-19S × HL	0.01 (0.00, 0.02)	0.017	0.01 (−0.01, 0.02)	0.079
**Interaction between FCoV-19S and eHEALS**				
FCoV-19S, 1-score increment	−1.51 (−1.86, −1.17)	<0.001	−1.18 (−1.51, −0.85)	<0.001
eHEALS, 1-score increment	0.14 (−1.11, 0.38)	0.273	0.09 (−0.14, 0.32)	0.451
FCoV-19S × eHEALS	0.02 (0.01, 0.03)	<0.001	0.01 (0.01, 0.02)	0.034
**Interaction between FCoV-19S and DDL**				
FCoV-19S, 1-score increment	−1.12 (−1.33, −0.91)	<0.001	−1.02 (−1.22, −0.82)	<0.001
DDL, 1-score increment	0.17 (0.02, 0.32)	0.026	0.03 (−0.11, 0.17)	0.652
FCoV-19S × DDL	0.01 (0.00, 0.02)	0.003	0.01 (0.00, 0.02)	0.016

Abbreviations: HRQoL, health-related quality of life; B, unstandardized regression coefficient; CI, confidence interval; FCoV-19S, fear of coronavirus disease 2019 Scale; HL, health literacy; eHEALS, eHealth literacy; DDL, digital healthy diet literacy. * Model 1: Simple linear regression model. ** Model 2: Adjusted for age, gender, marital status, occupation, social status, BMI, physical activity, and healthy eating.

## Data Availability

Data will be available on the reasonable request from the corresponding author.

## Supplementary Materials

The following are available online at https://www.mdpi.com/article/10.3390/ijerph18094929/s1, Table S1. Confounders associated with health-related quality of life among outpatients (N = 4348). Table S2. Spearman’s correlations (rho) among the studied variables (N = 4348). Table S3. Pearson’s correlations (rho) among fears of COVID-19, digital healthy diet literacy, eHealth literacy, and health literacy (N = 4348).

## Abbreviations

B	Unstandardized regression coefficient
DDL	Digital healthy diet literacy
eHEALS	eHealth Literacy Scale
FCoV-19S	Fear of COVID-19 Scale
HL	Health literacy
HLS-SF12	12-item short-form health literacy questionnaire
HRQoL	Health-related quality of life
OPD	Outpatient departments
S-COVID-19-S	Suspected COVID-19 symptoms
