# Supplementary material for: Negative Impact of Fear of COVID-19 on Health-Related Quality of Life Was Modified by Health Literacy, eHealth Literacy, and Digital Healthy Diet Literacy: A Multi-Hospital Survey
Source: Int J Environ Res Public Health. 2021 May 6;18(9):4929. doi: 10.3390/ijerph18094929 (PMC8124355; doi:10.3390/ijerph18094929)
Supplement: Supplementary file 1 [file ijerph-18-04929-s001.zip › ijerph-1193125-supplementary.pdf]

Effect Modification by Health Literacy, eHealth Literacy, and Digital Healthy Diet Literacy on the Association between Fear of COVID-19 and Health-related Quality of Life: A Multi-hospital Survey

Supplementary Materials

Table S1. Confounders associated with health-related quality of life among outpatients (*n* = 4348)..........2

Table S2. Spearman's correlations (rho) among the studied variables (n= 4348). 3

Table S3. Pearson's correlations (*r*) among fear of COVID-19, digital healthy diet literacy, eHealth literacy, and health literacy (n= 4348). 4

Table S1. Confounders associated with health-related quality of life among outpatients (*n* = 4348).

Variables	Bivariate Model B[a] (95% CI)	*P* value
**Age groups**		
< 60	0.00	
≥ 60	-12.00 (-13.24 to -10.76)	<0.001
**Gender**		
Women	0.00	
Men	0.42 (-0.68 to 1.52)	0.45
**Marital status**		
Never married	0.00	
Ever married	-7.93 (-9.31 to -6.56)	<0.001
**Education attainment**		
Secondary school or below	0.00	
High school	2.88 (1.41 to 4.36)	<0.001
College/university or higher	8.60 (7.28 to 9.91)	<0.001
**Occupation**		
Unemployed/ Dependents	0.00	
Employed	7.48 (5.79 to 9.18)	<0.001
**Ability to pay for medications**		
Very or fairly difficult	0.00	
Very or fairly easy	6.16 (5.08 to 7.25)	<0.001
**Social status**		
Low	0.00	
Middle or high	4.62 (3.32 to 5.91)	<0.001
**BMI, kg/m^2**		
Normal weight (BMI < 25.0)	0.00	
Overweight/obese (BMI ≥ 25.0)	-1.16 (-2.77 to 0.44)	0.16
**Comorbidity**		
No	0.00	
Yes	-5.01 (-6.18 to -3.84)	<0.001
**Suspected COVID-19 symptoms**		
No	0.00	
Yes	-5.83 (-6.90 to -4.75)	<0.001
**Smoking tobacco**		
Never, stopped, or smoke less	0.00	
Unchanged or smoke more	0.97 (-0.98 to 2.92)	0.33
**Drinking alcohol**		
Never, stopped, or drink less	0.00	
Unchanged or drink more	0.11 (-1.93 to 2.15)	0.92
**Physical activity**		
Never, stopped, or exercise less	0.00	
Unchanged or exercise more	11.21 (10.19 to 12.24)	<0.001
**Healthy eating**		
Less healthy	0.00	
Unchanged or healthier	8.15 (6.13 to 10.16)	<0.001

[a]B: Unstandardized regression coefficient

Table S2. Spearman's correlations (rho) among the studied variables (n= 4348).

Variables	Age	Gender	Marital status	Education	Occupation	Ability to pay	Social status	Comorbidity	BMI	S-COVID-19-S[a]	Smoking	Drinking	Eating behavior	Physical activity
Gender	.06													
Marital status	.21	-.03												
Education	**-.34**	.01	-.25											
Occupation	-.19	.02	.06	.16										
Ability to pay	-.21	-.03	-.06	.26	.17									
Social status	-.05	.00	.01	.24	.18	**.30**								
Comorbidity	.29	.08	.11	-.05	-.07	-.13	-.08							
BMI	.01	.06	.06	-.01	.01	.04	.03	-.01						
S-COVID-19-S[a]	.22	.01	.13	.05	-.07	-.12	-.01	**.50**	.01					
Smoking	.01	.17	.04	-.07	-.01	-.03	-.09	-.02	.01	-.05				
Drinking	.02	.15	.01	-.05	-.02	-.04	-.07	-.06	.02	-.08	**.66**			
Eating behavior	-.19	-.02	-.03	.07	.03	.09	-.02	-.17	-.02	-.14	.02	.01		
Physical activity	-.15	.04	-.12	.05	.04	.13	.01	-.14	-.04	-.26	.17	.19	.14	
FCoV-19S[b]	.01	-.06	.06	-.19	-.03	.01	-.06	-.15	-.01	-.21	.03	.06	.03	.06
Health literacy	-.23	-.02	-.18	.19	.08	.19	.18	**-.38**	.02	**-.34**	-.10	-.06	.05	.19
eHEALS[c]	-.21	-.03	-.16	.15	.10	.12	.13	**-.38**	-.01	**-.35**	-.11	-.09	.03	.16
DDL[d]	-.29	-.03	-.18	.22	.11	.22	.19	**-.37**	.02	**-.31**	-.11	-.06	.07	.19

[a]S-COVID-19-S: suspected coronavirus disease 2019 symptoms
[b]FCoV-19S: fear of coronavirus disease 2019 scale
[c]eHEALS: eHealth literacy
[d]DDL: digital healthy diet literacy

Table S3. Pearson's correlations (*r*) among fear of COVID-19, digital healthy diet literacy, eHealth literacy, and health literacy (n= 4348).

Variables	1	2	3	4
1. Fear of COVID-19 Scale, 1-score increment	1			
2. Health literacy, 1-score increment	0.04	1		
3. eHealth Literacy Scale, 1-score increment	0.09	**0.66**	1	
4. Digital healthy diet literacy, 1-score increment	0.01	**0.83**	**0.61**	1
